# Targeting PRMT5 in Adult T-Cell Leukemia/Lymphoma: Opportunities and Challenges

**DOI:** 10.3390/v18010094

**Published:** 2026-01-09

**Authors:** Kyle Ernzen, Amanda R. Panfil

**Affiliations:** Center for Retrovirus Research, Department of Veterinary Biosciences, The Ohio State University, Columbus, OH 43210, USA; ernzen.1@osu.edu

**Keywords:** ATLL, epigenetic, HTLV-1, PRMT5, therapy

## Abstract

Adult T-cell leukemia/lymphoma (ATLL) is an aggressive T-cell malignancy caused by persistent infection with human T-cell leukemia virus type 1 (HTLV-1). ATLL remains difficult to treat despite intensive chemotherapy, antiviral therapy, and hematopoietic stem cell transplantation. The limited durability of current treatment strategies highlights the need for mechanism-based therapeutic approaches. Protein arginine methyltransferase 5 (PRMT5) is a type II arginine methyltransferase that regulates transcription, RNA splicing, DNA damage responses, and immune signaling through symmetric dimethylation of histone and non-histone substrates. PRMT5 is frequently overexpressed across hematologic and solid tumors. Preclinical studies indicate that PRMT5 expression is elevated during HTLV-1-mediated T-cell transformation and that pharmacologic inhibition of PRMT5 selectively impairs the survival and transformation of infected T cells in vitro and in vivo. In this review, we highlight the current understanding of PRMT5 biology in cancer, summarize preclinical studies supporting PRMT5 as a therapeutic target in ATLL, and discuss key challenges to future clinical translation. We also discuss emerging approaches such as rational combination therapies and tumor-selective PRMT5 inhibitors as potential paths toward treatment for ATLL.

## 1. ATLL Background

Adult T-cell leukemia/lymphoma (ATLL) is an aggressive, non-Hodgkin’s malignancy caused by the oncogenic retrovirus human T-cell leukemia virus type 1 (HTLV-1) [1,2,3]. HTLV-1 infects an estimated 5–10 million people worldwide and is endemic in the Caribbean, southwestern Japan, Africa, South America, and the Pacific islands [4]. This retrovirus primarily infects CD4+ T cells, integrating its viral genome into the host chromosome and causing a persistent life-long infection of the host. The virus expresses multiple viral genes that drive the clonal proliferation of infected cells. Over time, the combination of genetic and epigenetic changes in the transformed cell can lead to ATLL development, providing a potential role for cellular factors to enhance pathogenesis over time [5]. Following a prolonged clinical latency period of several decades, approximately 5% of infected individuals will develop ATLL. This disease risk increases to 25% in individuals infected at birth [6,7]. Furthermore, ATLL is a highly aggressive and chemotherapy-resistant disease that is often fatal less than one year after diagnosis [8,9]. While many aspects of HTLV-1 biology are understood, the detailed mechanisms of HTLV-1-induced ATLL disease development remain poorly defined. Current therapies for ATLL patients include combination chemotherapy, antiviral agents, and allogeneic stem cell transplantation [10]. However, these treatments offer limited efficacy and are often associated with high relapse rates and poor long-term survival. The rapid progression of disease after onset, coupled with the lack of effective treatment regimens, highlights the critical need for novel, targeted treatment strategies for ATLL patients.

ATLL is clinically heterogeneous and is classified into four major subtypes: acute, lymphoma, chronic, and smoldering. Each of these subtypes has distinct clinical features and prognoses (reviewed in [11]). The acute and lymphoma subtypes are highly aggressive, characterized by rapid disease progression, high tumor burden, and overall poor survival (median overall survival of <1 year after diagnosis [10]). The acute form commonly presents with leukemic involvement, hypercalcemia, and widespread organ infiltration, whereas the lymphoma subtype is dominated by nodal and extranodal disease with minimal peripheral blood involvement [10]. In contrast, the chronic and smoldering subtypes follow a more indolent course, with lower tumor burden and longer survival (median overall survival time of ~4 years [12]). Notably, both indolent types of ATLL can evolve into the more aggressive subtypes over time [13]. The chronic subtype is defined by persistent lymphocytosis and variable organ involvement, while the smoldering subtype typically presents with limited skin or pulmonary disease and minimal systemic symptoms [13]. This clinical heterogeneity has important implications for therapeutic decision-making and underscores the need for subtype-specific and stage-adapted treatment strategies.

## 2. PRMT5 in Oncogenesis

Protein arginine methyltransferase 5 (PRMT5) is a central epigenetic factor with broad relevance to cellular oncogenesis. As a type II PRMT enzyme, PRMT5 regulates the transcription of key regulatory genes by symmetric dimethylation of arginine residues on histone proteins [14]. Because PRMT5 catalyzes symmetric dimethylarginine (SDMA) deposition, changes in global or substrate-specific SDMA levels provide a direct pharmacodynamic readout of PRMT5 target engagement and on-target pathway inhibition. PRMT5 methylation of histone and non-histone proteins has also been shown to affect splicing, signal transduction, and the DNA damage response (DDR) [14].

PRMT5 activity is typically executed as a multiprotein complex with methylosome protein 50 (MEP50), also known as WD repeat domain 77 (WDR77), which stabilizes PRMT5 and promotes efficient substrate engagement and catalysis [15]. In many contexts, PRMT5 functions as an anchor that links chromatin regulation, RNA metabolism, and signaling through compartment-specific substrate selection. Nuclear PRMT5–MEP50 preferentially impacts transcriptional programs and RNA processing, including methylation of histones (e.g., H4R3, H3R8) and splicing-associated factors [16,17]. In contrast, cytoplasmic PRMT5 more strongly interfaces with signaling networks by methylating non-histone substrates and forming functionally distinct protein interactions [18]. Shifts in PRMT5 localization driven by oncogenic signaling, post-translational modifications, or altered binding partners can rewire its substrate landscape and downstream phenotypes. This helps explain why PRMT5 can support diverse cancer hallmarks (growth, stress tolerance, immune evasion) across many tumor types.

Increased expression of PRMT5 is relevant to the pathogenesis of both hematologic and solid tumors, and in most cases, this upregulation is associated with poor patient survival. PRMT5 expression has been characterized to directly affect the overall size and grade of multiple cancer types, such as lung, breast, and hepatocellular carcinomas [19,20,21]. Furthermore, PRMT5 activity has been shown to promote lymph node metastasis and drug resistance in a wide array of different cancers [22,23,24,25,26,27]. Genetic alterations in PRMT5 genes are rare, and thus control of PRMT5 in cancer has emerged as an attractive therapeutic target [28,29,30,31,32].

Given the role of PRMT5 in several physiological processes critical to oncogenesis, this epigenetic factor exhibits substantial promise as a potent therapeutic target. For example, PRMT5 has multiple substrates in the DDR pathway, including flap endonuclease 1 (FEN1), tyrosyl-DNA phosphodiesterase 1 (TDP1), and Rad9 [33,34,35]. This indicates that PRMT5 inhibition could sensitize tumor cells to chemotherapy treatment or drugs that induce the DDR. This has already been demonstrated in both mixed lineage leukemia (MLL)-rearranged leukemia and acute myeloid leukemia (AML) [36,37]. PRMT5 activity has additionally been shown to aid in the self-renewal of cancer stem cells in both breast cancer and chronic myelogenous leukemia (CML) [38,39]. Due to the contribution of cancer stem cells in the development of drug resistance, targeting PRMT5 may also serve to mitigate the relapse potential from acquired therapeutic resistance. PRMT5 expression has been shown to enhance antitumor immunity by compromising immune cell activation, recruitment, and tumor recognition by indirectly repressing stimulator of interferon genes (STING) activation and type-1 interferon responses. These findings suggest that PRMT5 inhibitors (PRMT5i) may synergize with immune checkpoint therapies in unresponsive tumors, a notion that has already been illustrated in melanoma studies utilizing a PRMT5i and anti-programmed cell death protein 1 (PD1) treatment regimen [40,41]. Altogether, the notable roles of PRMT5 in the DDR, cancer stem cell renewal, and tumor immunity demonstrate strong precedence for the development of novel PRMT5i against the pathogenesis of solid and non-solid tumors.

## 3. Preclinical Evaluation of PRMT5 as a Therapeutic Target in ATLL

PRMT5 has emerged as a compelling therapeutic target across a wide range of malignancies, including leukemias and lymphomas [39,42,43,44,45]. Given the aggressive nature of ATLL and its poor response to conventional chemotherapy, there is an urgent need to identify novel and effective therapeutic strategies. Early work explored the potential of PRMT5 inhibition in the context of HTLV-1 infection and ATLL pathogenesis. Initial expression analyses revealed that PRMT5 RNA and protein levels are markedly elevated in a variety of HTLV-1-transformed and ATLL-derived cell lines, as well as in peripheral blood mononuclear cells (PBMCs) obtained from ATLL patients. PRMT5 overexpression was also observed throughout the course of HTLV-1-mediated T-cell transformation in vitro [46], suggesting PRMT5 may contribute to the early stages of leukemogenesis and could serve as a therapeutic vulnerability in HTLV-1-infected cells.

Follow-up studies have assessed the therapeutic relevance of PRMT5 inhibition using the small molecule inhibitor, EPZ015666. This next-generation peptide-competitive PRMT5 inhibitor was previously shown to have high specificity and potency in mantle cell lymphoma models [47] and has undergone clinical evaluation for multiple solid and other non-solid cancers [48,49]. Dose escalation experiments demonstrated that EPZ015666 induced robust, dose-dependent cytotoxicity in HTLV-1-infected and ATLL-derived cell lines, with little-to-no toxicity against naïve T-cells. This selectivity was further supported by increased expression of cleaved poly(ADP-ribose) polymerase (PARP) and the induction of apoptosis in EPZ015666-treated cells. Furthermore, in a co-culture model of HTLV-1-mediated transformation using primary PBMCs and irradiated virus producer cells, weekly administration of EPZ015666 completely blocked cellular immortalization. This indicates that PRMT5 activity is essential for HTLV-1-driven T-cell transformation in vitro [50].

Building upon in vitro findings, the therapeutic potential of PRMT5 inhibition has also been evaluated in vivo using xenograft mouse models. In immunocompromised NSG (NOD.Cg-PrkdcscidIl2rgtm1Wjl/SzJ) xenograft mice inoculated with either HTLV-1-transformed (SLB-1) or ATLL-patient-derived (ATL-ED) cell lines, treatment with EPZ015666 significantly reduced tumor growth, lowered serum interleukin-2 receptor alpha chain (IL-2Rα) levels—a surrogate marker of HTLV-1-infected cell burden—and improved overall survival [51]. Similar therapeutic benefit was observed in an NSG humanized immune system mouse model. These NSG-based humanized mice support engraftment of human T cells, but retain profound defects in adaptive immunity, providing a controlled human-immune context for modeling HTLV-1 infection and disease development. In this model, EPZ015666 administration similarly led to marked survival improvements following HTLV-1 infection and disease progression [50]. Collectively, these initial findings demonstrate that PRMT5 inhibition selectively impairs the survival and transformation of HTLV-1-infected cells both in vitro and in vivo.

In a recent study by Ichikawa et al. [52], the authors examined PRMT5 inhibition in ATLL cells characterized by low expression of N-myc downstream-regulated gene 2 (NDRG2), a tumor suppressor frequently downregulated in ATLL and other cancers [53,54]. NDRG2 is known to regulate the recruitment of protein phosphatase 2A (PP2A), and its loss leads to aberrant activation of the phosphoinositide 3-kinase/protein kinase B (PI3K/AKT) and nuclear factor kappa B (NF-κB) signaling pathways, thereby enhancing cellular transformation in HTLV-1-infected cells [55,56]. Notably, NDRG2 downregulation also promotes hyperphosphorylation and cytoplasmic translocation of PRMT5, leading to enhanced interaction with the molecular chaperone heat shock protein 90 alpha (HSP90A), a known facilitator of PRMT5 function [57]. Ichikawa et al. demonstrated that shRNA-mediated knockdown of PRMT5 in NDRG2-deficient ATLL cells significantly reduced cell proliferation and led to degradation of AKT and NF-κB essential modulator (NEMO), both HSP90A client proteins. Furthermore, NDRG2-low ATLL cell lines and patient-derived cells exhibited heightened sensitivity to PRMT5 inhibitor (PRMT5i) treatment, suggesting that NDRG2 expression may serve as a predictive biomarker for therapeutic response [52]. These findings highlight the mechanistic complexity of PRMT5 signaling in HTLV-1-infected cells and underscore its therapeutic relevance, particularly in molecularly defined subsets of ATLL.

**Figure 1:**
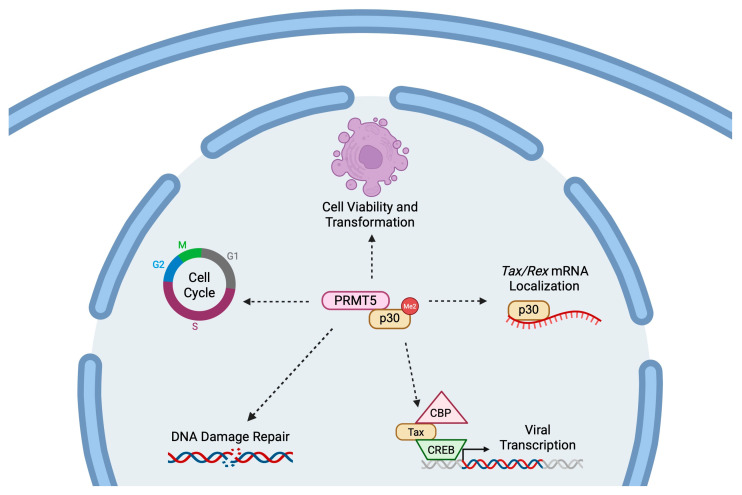
**Hypothetical role of PRMT5 inhibition in HTLV-1-infected cells.** PRMT5 interacts with the HTLV-1 accessory protein p30 and is proposed to influence multiple cellular and viral processes relevant to HTLV-1–mediated T-cell transformation. Through this interaction, PRMT5 may contribute to the regulation of cell cycle progression, DNA damage repair pathways, and overall cell viability and transformation. PRMT5–p30 activity may also be linked to p30-mediated control of Tax and Rex mRNA localization and modulation of viral transcription through the Tax–CREB–CBP transcriptional complex. Dashed arrows indicate proposed or indirect regulatory relationships that remain under investigation. Created in BioRender. Panfil, A. (2026) https://BioRender.com/a82o9ly.

These preclinical studies have established a strong rationale for further investigation of PRMT5 as a therapeutic target in ATLL and provide a foundation for future translational and clinical efforts. Additional studies will be necessary to determine the precise role of PRMT5 in promoting the transformation and pathogenesis of HTLV-1-infected cells. Early work indicated that PRMT5i administration led to enhanced viral gene expression in HTLV-1-infected and ATLL-derived cell lines [46]. Proteomics studies have further demonstrated that PRMT5 is a binding partner of the viral accessory protein p30 [58]. p30 functions as a negative post-transcriptional regulator of viral gene expression and Tax transcriptional activity [59,60]. The p30 protein has also been implicated in the regulation of several key cellular processes, including cell cycle progression, the DNA damage response, and T-cell proliferation [61,62,63]. Future investigation of the interaction between PRMT5 and p30 may provide a rationale for the therapeutic efficacy of PRMT5i against HTLV-1-mediated T-cell transformation and disease progression (Figure 1).

## 4. Challenges to PRMT5 Inhibition

Despite promising preclinical results, several challenges remain in establishing PRMT5 inhibition as a viable therapeutic strategy for ATLL. PRMT5 plays an essential role in normal T-cell biology, promoting IL-2 production and cell proliferation. Inhibition of PRMT5 has been shown to impair memory T-cell expansion, especially within Th1 cell subsets [64]. These immunomodulatory effects raise concerns about the long-term safety and tolerability of PRMT5 inhibitors in ATLL patients, who may already have compromised immune function due to disease or concurrent treatments. PRMT5 expression is also crucial for many physiological processes, including motor function, hematopoiesis, and maintenance of the central nervous system [65,66,67]. PRMT5 is especially essential during early development, where complete loss of PRMT5 expression induces embryonic lethality in mice [68]. Furthermore, phase 1 clinical trials for patients with solid tumors resulted in numerous adverse events, including anemia, thrombocytopenia, and neutropenia following administration of first-generation PRMT5i [69,70,71].

Another key obstacle in the clinical development of small-molecule therapies, including PRMT5i, is the emergence of acquired resistance. A recent study by Long et al. investigated resistance mechanisms to PRMT5 inhibition in mantle cell lymphoma (MCL) [72]. Using a patient-derived xenograft (PDX) model, the authors demonstrated that tumors resistant to PRMT5i exhibited significantly diminished responses and worse survival outcomes compared to vehicle-treated controls. Transcriptomic analyses of resistant cell lines revealed upregulation of the mammalian target of rapamycin (mTOR) pathway. Targeting this adaptive mechanism, the authors found that combining PRMT5i with the mTORC1 inhibitor temsirolimus produced synergistic anti-proliferative effects in vitro and improved survival in vivo. These findings not only highlight a plausible mechanism of resistance but also provide a rationale for combination therapy as a strategy to enhance and sustain PRMT5i efficacy.

Despite decades of research, ATLL remains a highly aggressive malignancy with limited curative treatment options. Current therapeutic approaches, such as intensive chemotherapy followed by allogeneic hematopoietic stem cell transplantation, are associated with significant toxicity and are often unsuitable for immunocompromised or elderly patients. Moreover, ATLL frequently exhibits resistance to standard chemotherapy treatments [73]. The combination of azidothymidine (AZT), a well-known HIV-1 antiviral, and interferon-alpha (IFNα) has shown clinical benefit in select patient populations [74,75,76], but its therapeutic effects are often transient, underscoring the need for new strategies. Several PRMT5 inhibitors are currently in clinical development for various hematologic and solid tumors, often in combination with chemotherapy, small molecule inhibitors/targeted agents, or immunotherapies (reviewed in [77,78]). While PRMT5 inhibition alone may not be sufficient to achieve a durable response in ATLL, its integration into rational combination regimens—guided by mechanistic insights and molecular biomarkers such as NDRG2—may improve therapeutic efficacy. Continued research into the role of PRMT5 in HTLV-1 infection and ATLL progression will be essential to inform optimal treatment strategies and support the translation of PRMT5-targeted therapies into clinical practice.

## 5. Future Directions for PRMT5 Inhibition in ATLL Therapy

PRMT5 is a multifunctional arginine methyltransferase that targets a wide array of histone and non-histone proteins involved in regulating gene transcription, cell signaling pathways, DNA damage repair, mRNA splicing, and immune regulation. When PRMT5 is overexpressed, dysregulation of these pathways can promote oncogenic transformation, leading to enhanced pathogenesis and lower patient survival rates [41]. Therefore, PRMT5 inhibition alone is unlikely to provide a durable therapeutic benefit in ATLL. This highlights the need to identify complementary oncogenic pathways that can be co-targeted to enhance efficacy and prevent resistance.

Several studies provide strong support for combination-based PRMT5 inhibition strategies. Long et al. demonstrated that dual targeting of PRMT5 and mTOR overcomes PRMT5i resistance in MCL cells, both in vitro and in vivo [72]. Similar synergistic activity has been observed in glioblastoma, where combined PRMT5 and mTOR inhibition produced superior antitumor effects compared with either inhibitor alone [79]. Notably, mTOR inhibition was shown to increase PRMT5 activity in glioblastoma cells, providing a mechanistic rationale for this synergy. Given that both PRMT5 and mTOR are upregulated in ATLL patient cells [46,80], evaluating this combinatorial approach in ATLL models represents a compelling avenue for future investigation.

Additional combination strategies further underscore the therapeutic versatility of PRMT5 inhibitors. In a preclinical study by Brown et al., the authors found that co-administration of PRMT5 and B-cell lymphoma 2 (BCL-2) inhibitors produced synergistic antitumor activity in an ibrutinib-resistant MCL PDX mouse model. Importantly, the PRMT5/BCL-2 inhibitor combinatorial treatment had synergistic antitumor activity without inducing host toxicity [81], illustrating the potential of PRMT5i to reduce oncogenesis and overcome acquired drug resistance. Moreover, a study by Kim et al. demonstrated that combining PRMT5i with an anti-PD1 antibody significantly reduced tumor burden and improved survival in an immunocompetent murine melanoma model. Mechanistically, the PRMT5 antagonism augmented interferon levels, chemokine production, and MHC class I expression, thereby improving tumor cell recognition [40]. Together, these findings suggest that PRMT5i may synergize effectively with immunotherapeutic approaches and could be well-suited for T-cell malignancies such as ATLL. These preclinical studies provide a strong rationale that utilizing PRMT5i as part of rational combination therapies may improve antitumor efficacy and limit acquired drug resistance.

To reduce the risk of harmful off-target toxicities associated with systemic PRMT5 inhibition, considerable effort is now focused on developing tumor-selective PRMT5 inhibitors [82,83,84,85]. One promising strategy exploits the loss of methylthioadenosine phosphorylase (MTAP). MTAP is a key enzyme in the methionine salvage pathway and is solely responsible for degrading methylthioadenosine (MTA), a nucleoside that weakly binds to and inhibits PRMT5 activity. MTAP deletions occur in approximately 10–15% of human cancers, including a subset of ATLL cases [86,87,88,89]. MTAP-null tumors typically exhibit a substantial accumulation of MTA, leading to abnormally high levels of PRMT5-MTA complexes [90,91]. These complexes can be selectively targeted by MTA-cooperative PRMT5 inhibitors. Several clinical-stage compounds, such as AMG193, TNG908, and MRTX1719, have been designed and utilized to take advantage of this type of small molecule-mediated precision therapy [82,83,85]. MRTX1719 was administered following disease onset in xenograft mice inoculated with Lu-99 cells, a MTAP-CDKN2A-deleted human lung cancer cell line. MRTX1719-treated mice achieved upwards of 86% tumor growth inhibition and significantly reduced plasma SDMA levels, a pharmacodynamic marker of PRMT5 activity [82]. Similarly, TNG908 demonstrated potent efficacy in vivo across multiple MTAP-null tumor models, including lung, colorectal, and glioblastoma cancers. Administration of TNG908 in xenograft mice inoculated with MTAP-null tumor cells from each of these respective cancer types resulted in significant antitumor activity and a substantial reduction in SDMA levels [83]. Although these MTA-cooperative PRMT5 inhibitors have not yet been evaluated against ATLL, their tumor-selective activity suggests substantial therapeutic promise for future application in MTAP-deficient ATLL subsets.

PRMT5 inhibition initiated at the time of disease onset significantly reduces tumor burden and improves survival in HTLV-1-infected xenograft and humanized mouse models [50]. While these findings establish PRMT5 as a promising therapeutic target for ATLL, additional in vivo studies are required to define the optimal timing, durability, and translational potential of PRMT5-directed therapies. The therapeutic efficacy of PRMT5 inhibitors against ATLL and other cancer subtypes is summarized in Table 1. Ongoing and future work should evaluate the efficacy of PRMT5 inhibitor administration in animal models prior to overt disease development, with the goal of determining whether early PRMT5 blockade can prevent or attenuate ATLL pathogenesis. This approach is supported by the in vitro evidence that PRMT5 inhibition blocks HTLV-1-mediated T-cell transformation [50].

## 6. Conclusions

ATLL remains a devastating and highly aggressive T-cell malignancy. Existing treatment strategies, such as chemotherapy, hematopoietic stem cell transplantation, and AZT-IFNα administration, provide limited durability and are frequently followed by relapse. This underscores the need for new mechanism-based therapeutic approaches. Accumulating preclinical evidence supports PRMT5 as a key epigenetic regulator of HTLV-1-mediated T-cell transformation and ATLL pathogenesis. Pharmacologic inhibition of PRMT5 selectively impairs the survival of HTLV-1-infected and ATLL-derived cell lines, blocks virus-driven T-cell transformation in vitro, and improves disease outcomes in multiple in vivo models. In the future, further mechanistic studies are needed to define how PRMT5 supports viral gene function and host transcriptional programs and to identify biomarkers that predict therapeutic response. Given the pleiotropic roles of PRMT5 in oncogenic signaling, immune recognition, and DNA damage responses, PRMT5 inhibition is unlikely to be maximally effective as a monotherapy. Integration of PRMT5 inhibitors into rational combination regimens, such as co-targeting mTOR signaling or immune checkpoint pathways, may enhance therapeutic durability and overcome resistance. Additionally, the timing of PRMT5 inhibitor administration may help clarify the potential for this drug to delay or attenuate ATLL development. Collectively, the extensive body of work implicating PRMT5 in diverse cancers and disease contexts establishes this epigenetic factor as a promising and biologically grounded therapeutic target. When integrated with emerging studies in the HTLV-1 field, these findings provide a strong framework for the future clinical translation of PRMT5-directed strategies in ATLL.

## Figures and Tables

**Table 1:** **Therapeutic impact of PRMT5 inhibitors against various malignancies.**

Primary Compound	Mode of Inhibition	Disease	Secondary Compound	Primary Impact	Reference Number
GSK3186000A	Direct	AML	PARP inhibitor Olaparib	PRMT5 and PARP inhibitors display synergy against AML colony formation	[36]
EPZ015666	Direct, SAM-uncompetitive	MLL-r	DOTL1 inhibitor EPZ004777	PRMT5i and DOTLi synergistically repress MLLr cell proliferation	[37]
GSK3326595	Direct, SAM-uncompetitive	Melanoma	Anti-PD1 antibodies	PRMT5i and anti-PD1 antibodies synergistically reduced tumor size in grafted C57BL/6 mice in vivo	[40]
CMP5	Direct, SAM-competitive	HTLV-1	None	PRMT5i selectively decreased HTLV-1 cell viability and proliferation	[46]
EPZ015666	Direct, SAM-uncompetitive	MCL	None	PRMT5i caused dose-dependent antitumor activity in xenograft mice in vivo	[47]
EPZ015666	Direct, SAM-uncompetitive	HTLV-1	None	PRMT5i improved overall survival of HTLV-1-infected humanized and xenograft mice in vivo	[50]
HLCL61	Direct, SAM-competitive	ATLL	None	PRMT5i selectively decreased viability of ATL patient cells	[52]
PRT-808	Direct, SAM-competitive	MCL	mTOR inhibitor Temsirolimus	PRMT5i and mTORi synergistically improved overall survival of PRMT5i-resistant mice in vivo	[72]
PRT-382	Direct, SAM-competitive	MCL	BCL-2 inhibitor Venetoclax	PRMT5i and BCL-2i synergistically improved overall survival of ibrutinib-resistant mice in vivo	[81]
MRTX1719	Direct, MTA-dependent	*MTAP/CDKN2A*-deleted lung cancer	None	PRMT5i induced 86% tumor growth inhibition in xenograft mice in vivo	[82]
TNG908	Direct, MTA-dependent	*MTAP*-null NSCLC, CRC, and GBM	None	PRMT5i demonstrated selective antitumor activity in xenograft mice in vivo	[83]

Legend: AML (acute myeloid leukemia), ATLL (adult T-cell leukemia/lymphoma), BCL-2 (B-cell lymphoma-2), CRC (colorectal cancer), DOTL1 (disruptor of telomeric silencing 1-like), GBM (glioblastoma), HTLV-1 (human T-cell leukemia virus type-1), MCL (mantle cell lymphoma), MLLr (mixed lineage leukemia-rearranged), MTA (methylthioadenosine), mTOR (mechanistic target of rapamycin kinase), NSCLC (non-small cell lung cancer), PARP (poly ADP-ribose polymerase), PD1 (programmed cell death protein 1), PRMT5 (protein arginine methyltransferase 5), and SAM (S-adenosyl methionine).

## Data Availability

No new data were created or analyzed in this study.

## Abbreviations

The following abbreviations are used in this manuscript:

AML	Acute myeloid leukemia
ATLL	Adult T-cell leukemia/lymphoma
AZT	Azidothymidine
BCL-2	B-cell lymphoma 2
CML	Chronic myelogenous leukemia
CRC	Colorectal cancer
DDR	DNA damage response
DOTL1	Disruptor of telomeric silencing 1-like
FEN1	Flap endonuclease 1
GBM	Glioblastoma
HSP90A	Heat shock protein 90 alpha
HTLV-1	Human T-cell leukemia virus type 1
IL-2Rα	Interleukin-2 receptor alpha chain
IFNα	Interferon-alpha
MCL	Mantle cell lymphoma
MEP50	Methylosome protein 50
MLL	Mixed lineage leukemia
MLLr	Mixed lineage leukemia-rearranged
MTA	Methylthioadenosine
MTAP	Methylthioadenosine phosphorylase
mTOR	Mammalian target of rapamycin
NEMO	NF-κB essential modulator
NF-κB	Nuclear factor kappa B
NDRG2	N-myc downstream-regulated gene 2
NSCLC	Non-small cell lung cancer
NSG	NOD.Cg-Prkdc^scid^Il2rg^tm1Wjl^/SzJ
PARP	Poly (ADP-ribose) polymerase
PBMC	Peripheral blood mononuclear cell
PD1	Programmed cell death protein 1
PDX	Patient-derived xenograft
PP2A	Protein phosphatase 2A
PRMT5	Protein arginine methyltransferase
PRMT5i	Protein arginine methyltransferase inhibitor
SAM	S-adenosyl methionine
SDMA	Symmetric dimethylarginine
STING	Stimulator of interferon genes
TDP1	Tyrosyl-DNA phosphodiesterase 1
WDR77	WD repeat domain 77
